# Fragment‐Based Screening of Heterocyclic Electrophiles Identified Acrylamide Bioisoteres for Covalent KRas^G12C^ Inhibitors

**DOI:** 10.1002/ardp.70342

**Published:** 2026-09-21

**Authors:** Nikolett Péczka, Aaron Keeley, Péter Ábrányi‐Balogh, Zoltán Orgován, László Petri, Emma K. Grant, Chad Quinn, Soma Csanády, Ivan Randelovic, József Tóvári, Jacob Bush, György M. Keserű

**Affiliations:** ^1^ Medicinal Chemistry Research Group and National Drug Discovery and Development Laboratory HUN‐REN Research Centre for Natural Sciences Budapest Hungary; ^2^ Department of Organic Chemistry and Technology Budapest University of Technology and Economics Budapest Hungary; ^3^ GlaxoSmithKline Stevenage UK; ^4^ Department of Experimental Pharmacology and the National Tumor Biology Laboratory National Institute of Oncology Budapest Hungary

**Keywords:** electrophile, fragment screen, heterocycle, KRas, targeted covalent inhibitor

## Abstract

Covalent fragment approaches gained increasing attention in medicinal chemistry. Our covalent heterocyclic fragment library (Covalent MiniFrags) consisting of five and six membered heterocycles was screened against the oncogenic target KRas^G12C^. First, we detected the labeling of cysteine residues by Ellman's free thiol assay. Primary hits were characterized in a GSH reactivity assay and next we confirmed covalent labeling by RapidFire MS. Fragment hits with the best potential and selectivity were combined in silico with the quinazoline core of ARS‐1620, and the best candidate was chosen for synthesis. Binding of the heterocyclic ARS derivative was confirmed via intact MS and HSQC NMR measurements. Its functional activity was characterized by nucleotide exchange assay and cell viability assay on cell lines expressing KRas^G12C^. Our results support that screening of the heterocyclic electrophilic fragment library could provide viable chemical starting points for the development of targeted covalent inhibitors.

## Graphical abstract

We screened acrylamide bioisosteres against KRas^G12C^ and prioritized primary hits by their GSH reactivity, labeling efficiency and specificity. The most promising fragment was combined with a KRas‐binding scaffold. Covalent binding, inhibitory activity and selectivity were confirmed via MS, NMR, biochemical and cell viability assays. We suggest that this workflow might provide reasonable starting points for targeted covalent inhibitor development.

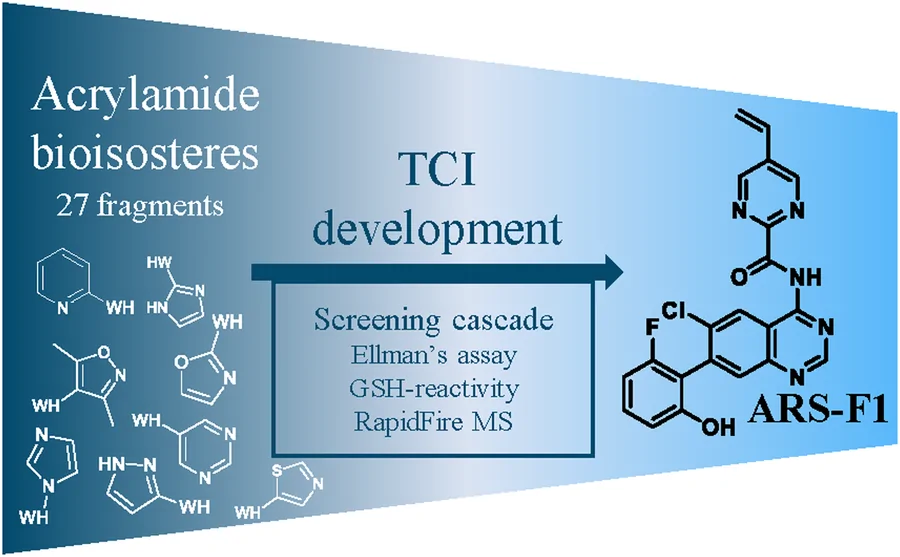

## 1 Introduction

Nitrogen containing heterocycles are common building blocks of FDA‐approved drugs. Pyridine, pyrimidine, pyrazole, imidazole and thiazole are among the 12 most frequently appearing nitrogen heterocycles usually providing favorable physicochemical properties and enhanced binding to the targeted site [1, 2, 3, 4, 5]. Their electron‐withdrawing properties, can be exploited to activate electrophilic substituents, while the heterocyclic core can contribute to ligand binding. Previously we showed that heterocycles equipped with small electrophilic substituents (Covalent MiniFrags), like halogen atoms, vinyl, nitrile or ethynyl groups cover a wide range in reactivity and they can be used as tunable electrophiles for cysteine labeling [6, 7, 8, 9]. Compared with larger warheads such as maleimides, acrylamides and vinyl sulfones, these substituents are expected to have a limited impact on binding properties of the parent scaffold. In addition, vinyl heterocycles are much less prone to retro‐Michael addition compared to maleimides and acrylamides that provides better stability to their conjugates [10, 11, 12, 13]. These advantages nominate heterocyclic electrophiles for fragment‐based covalent inhibitor discovery as exemplified by exploring allosteric and cryptic binding sites, demonstrated with HDAC8 [8], and by replacing the acrylamide warhead of ibrutinib with heterocyclic electrophiles as bioisosteres [14].

**Figure 1:**
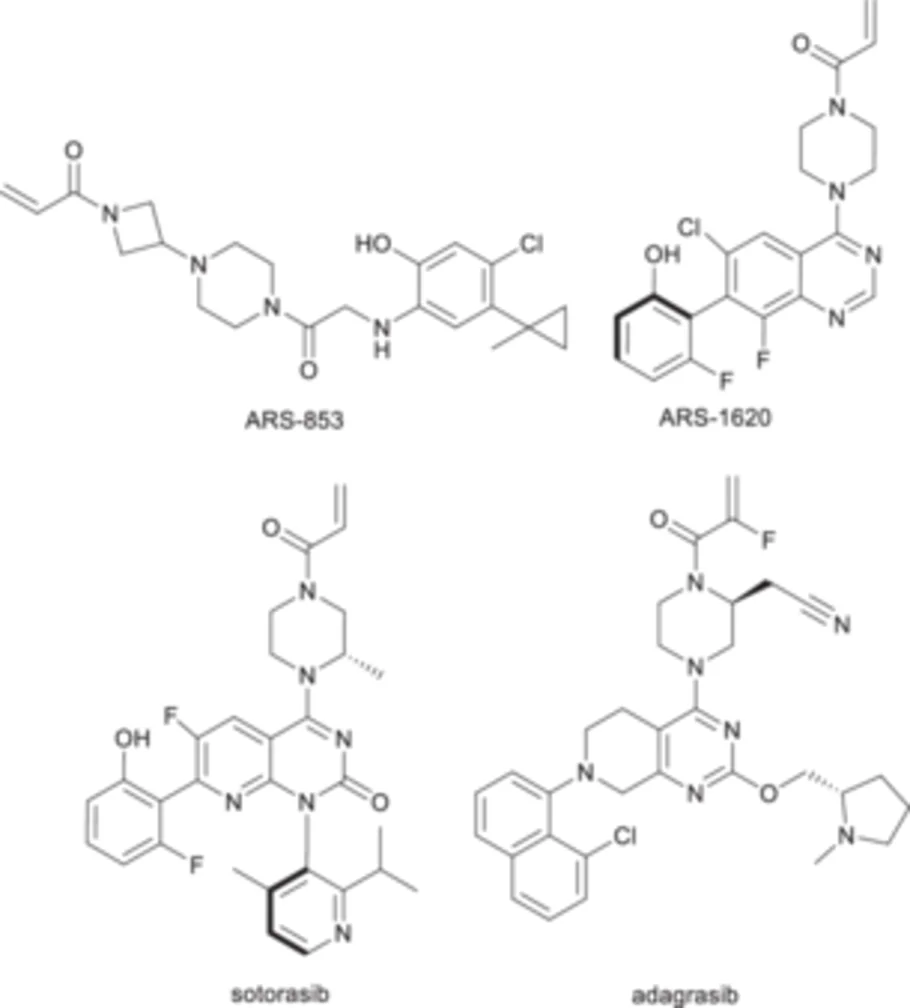
KRas^G12C^ inhibitors.

KRas had long been considered an undruggable target until the identification of Switch‐II pocket and the development of mutant‐selective covalent ligands targeting oncogenic C12 mutation. Subsequent optimization led to ARS‐853 [15] (Figure 1), a potent covalent inhibitor with and cellular activity. ARS‐853 binds to the Switch II pocket (S‐IIP) of KRas through the formation of a covalent bond with C12, thereby stabilizing the protein in its inactive, GDP‐bound state. However, its poor pharmacokinetic properties prevented its further development. Optimization of ARS‐853 by replacing its flexible core with a more rigid quinazoline scaffold led to the first in vivo active KRas^G12C^ inhibitor, ARS‐1620 [16] (Figure 1). This compound exhibited a more favorable orientation of the acrylamide warhead toward C12 and formed enhanced interactions with H95 and T96 that provided increased binding affinity to KRas. The optimization of these early inhibitors ultimately enabled the development of the clinically approved KRas^G12C^ inhibitors sotorasib [17] and adagrasib [18] (Figure 1) demonstrating that KRas^G12C^ is a druggable and clinically validated therapeutic target [19, 20, 21]. The Switch‐II pocket is conformationally dynamic, and currently approved inhibitors preferentially bind the inactive, GDP‐bound state, making their activity dependent on nucleotide cycling and the availability of the tractable protein conformation. Productive covalent inhibition requires a carefully balanced combination of noncovalent recognition, appropriate warhead orientation, and controlled cysteine reactivity. Heterocyclic electrophiles may provide opportunities to expand the limited warhead space, tune covalent reactivity, enhance noncovalent interactions, improve physicochemical properties, and explore alternative binding geometries facilitating the design of next generation KRas^G12C^ inhibitors.

Here we aim to extend the scope of the suitable KRas^G12C^ warheads and investigate whether heterocyclic covalent fragments provide a viable starting point for developing novel inhibitors. The first *in vivo* active KRas^G12C^ inhibitor ARS‐1620 contains an atropisomeric quinazoline core. However, later it turned out that the atropisomerism might be released and screening a diverse set of warheads on the piperazine attached to the quinazoline can lead to potent KRas^G12C^ covalent binders and inhibitors [22]. Therefore, we aimed to equip heterocyclic warheads to the non‐atropisomeric ARS‐type quinazoline core. Potential heterocyclic warheads were prioritized by screening a 27‐membered electrophilic fragment library of diverse 5‐ and 6‐membered heterocyclic acrylamide bioisosteres against KRas^G12C^. Further characterization of the hits nominated a heterocyclic warhead that was introduced to an ARS‐type KRas^G12C^ inhibitor core. We envisaged the substitution of the piperazine ring equipped usually with an acrylamide type Michael acceptor warhead to an electrophilic heterocycle leading to a new ARS derivative.

## 2 Results and Discussion

### 2.1 Screening of the Heterocyclic Fragments Against KRas^G12C^

**Figure 2:**
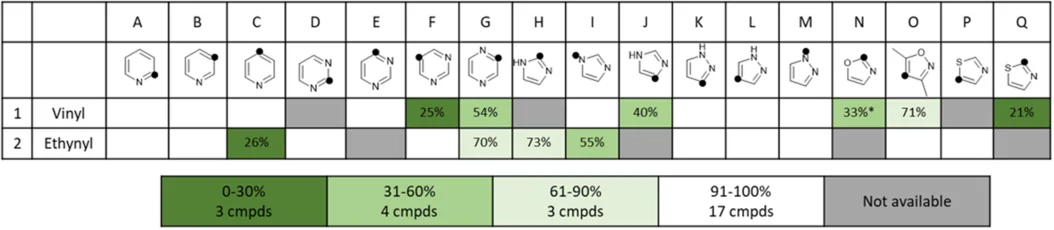
Colorimetric free thiol assay screen of the heterocyclic library. Black dots represent the position of the substituent; the percentages are the free thiol ratio (FTR). ARS‐853 was used as the positive‐ and dimethyl sulfoxide (DMSO) as the negative control. The compound marked with * this fragment have a phenyl substituent at Position 5.

First, we detected labeling of cysteine residues by a high throughput colorimetric free thiol assay [23, 24] that relies on the reaction between the available thiol groups and 5,5′‐dithiobis(2‐nitrobenzoate) (DTNB), and measures total remaining free thiol content. By screening 27 fragments in a hundredfold excess, we identified 5 hits (Figure 2 and Supporting Information: Table S1) that labeled more than 60% of the cysteines in KRas^G12C^. In addition to the oncogenic mutation at G12, chemoproteomic studies indicated that highly reactive iodoacetamide derivatives labeled cysteines C51, C80, C118 of the wild type KRas under reducing conditions. Under physiological conditions, however, only C118 is available for labeling [25, 26]. Considering cysteines available in KRas^G12C^, C118 is located on the surface, so despite its higher accessibility it is mostly protonated and therefore its reactivity against mild electrophiles is limited. C51 and C80 is natively buried deeply within the hydrophobic core of KRas suggesting limited accessibility [27]. In contrast, C12 can be found in a pre‐formed binding pocket, and its pK_a_ is depressed (pK_a_~7.6) [28], providing favorable accessibility and the highest intrinsic reactivity among the four cysteines. Acrylamides and vinyl heterocycles have mild reactivity, and acrylamide substituted KRas^G12C^ inhibitors (ARS‐853 and ARS‐1620) [15, 16] was proven to label selectively C12 in chemoproteomics experiments. Based on these arguments, we considered C12 the cysteine most likely to be labeled by our set of electrophile heterocycles. Thus, we assumed that fragment hits labeling more than half of the available cysteines might target the oncogenic mutation G12C with a high probability. In addition, we used ARS‐853 that labels only C12 as reference to analyze the labeling efficiency of heterocyclic fragments. Hits were identified as two 6‐membered and three 5‐membered heterocycles equipped with vinyl (**F1**, **J1**, **N1** and **Q1**), and ethynyl (**C2**) substituents. Next, we have characterized them in a glutathione reactivity assay in the presence of 20 equiv. GSH (Table 1) and found that **F1**, **J1** and **N1** have mild reactivity (*t*_1/2_ > 48 h) compared to the ethynyl derivative **C2** and to **Q1** in that the sulfur atom might increase reactivity with its participation in the conjugated electron system.

**Table 1:** Colorimetric free thiol assay (FTR – free thiol ratio), cell viability (H1792 cell line, in two concentrations) and glutathione (GSH)‐reactivity results of the best 5 compounds. In case of cell viability 0.00 is the value for the positive control producing dead cells, and 1.00 is the value for negative/untreated control. N.D.: not determined. ARS‐853 as commercially available KRas^G12C^ inhibitor was selected as reference.

Compound	FTR	25 µM	2.5 µM	GSH reactivity (t_1/2_)
**C2**	26%	0.98	0.98	2.40 h
**Q1**	21%	−0.01	0.01	2.70 h
**F1**	25%	0.00	0.02	> 48 h
**N1**	33%	0.01	0.77	> 48 h
**J1**	40%	0.00	0.12	> 48 h
**ARS‐853**	N.D.	0.6	0.78	N.D.

Next, we performed RapidFire MS measurements to prove covalent labeling of **F1**, **J1**, and **N1** on KRas^G12C^ and three benchmark proteins: lysozyme (eight cysteines are in disulfide bridges), myoglobin (no cysteine residues in the sequence) and carbonic anhydrase II (one available cysteine, C206) (Table 2). In case of lysozyme and myoglobin we observed very limited labeling (1‐4%) that probably could be attributed to minor lysine labeling [12]. Carbonic anhydrase was labeled presumably on its single cysteine in 7%–14%. In contrast, KRas^G12C^ was labeled with higher efficiency for **N1** and **F1**, and **F1** showed the highest labeling efficiency coupled with the highest selectivity over benchmark proteins. Site specific assignment was not performed at this stage.

**Table 2:** Average labeling values (%) resulted from RapidFire MS measurements of the active compounds on KRas^G12C^, lysozyme (Lys), myoglobin (Myo), and carbonic anhydrase II (C.A.).

Compound	KRas^G12C^	Lys	Myo	C.A.
**F1**	39.1	4.3	3.5	13.9
**N1**	19.8	4.5	3.0	10.7
**J1**	7.4	1.1	1.6	7.3

Considering its limited GSH reactivity, but best KRas^G12C^ labeling and good cytotoxicity, we prioritized **F1** for the design of a targeted covalent KRas^G12C^ inhibitor.

### 2.2 Design and Synthesis of Targeted Covalent Inhibitor

**Figure 3:**
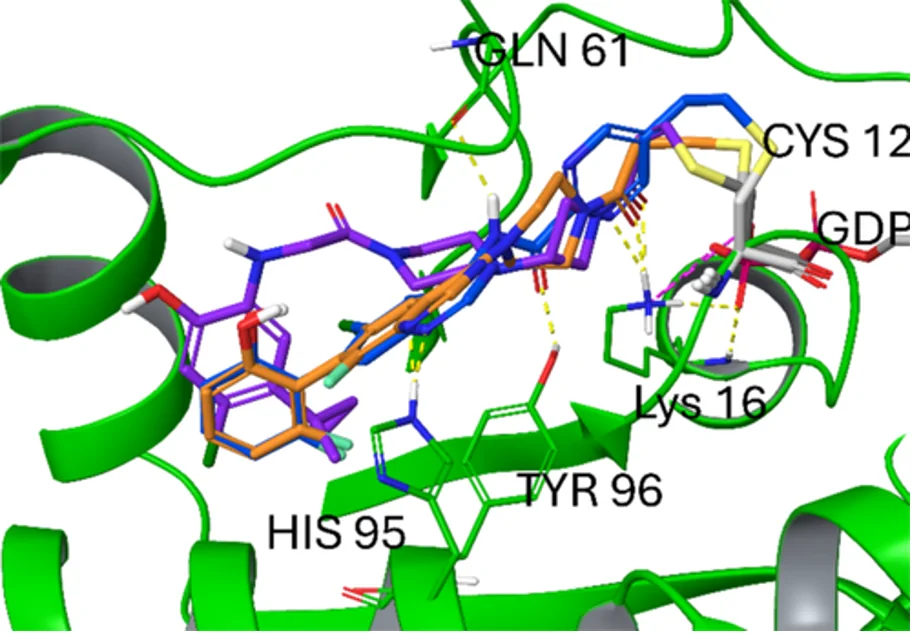
Modeled binding mode of **ARS‐F1** (blue) within the KRas^G12C^–ARS‐1620 complex X‐ray structure (PDB ID: 5V9U) with ARS‐853 (purple) and ARS‐1620 (orange). Relevant amino acids are highlighted as green sticks.

**F1** was then attached to the ARS‐1620 quinazoline core through an amide linker in silico. The amide linker was chosen by synthetic considerations. The designed compound was docked into the KRas^G12C^ X‐Ray structure complexed with ARS‐1620 (PDB: 5V9U). The binding mode of **ARS‐F1** was found to be similar (heavy atom RMSD = 0.676 Å) to the experimental pose of ARS‐1620 (Figure 3) that nominated this compound for synthesis.

**Scheme 1:**
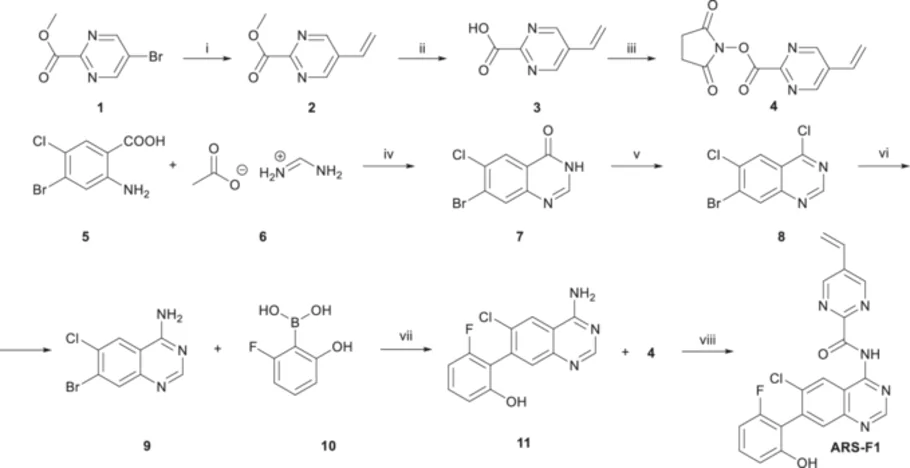
Synthesis of the heterocyclic warhead (**4**) and compound **ARS‐F1**. (i) tributyl(vinyl)stannane, Pd(PPh_3_)_2_Cl_2_, THF, 110°C, 2 h, 80%; (ii) HCl, H_2_O, 60°C, 6 days, 95%; (iii) TSTU, DIPEA, MeCN, rt, 30 min, 15%; (iv) EtOH, reflux, 16 h, 92%; (v) POCl_3_, DIPEA, 16 h, reflux, 82%; (vi) NH_3_/THF, rt, 1 h, 95%; (vii) RuPhos‐Pd‐G3, SPhos, Na_2_CO_3_, dioxane‐water, 80°C, 1 h, 97%; (viii) DIPEA, dioxane, 90°C, 16 h, 15%.

The heterocyclic warhead was synthesized from methyl 5‐bromopyrimidine‐2‐carboxylate (**1**) by vinylation (**2**) followed by ester hydrolysis leading to **3**. For the activation of the free acid, we transformed **3** to **4** using TSTU (*O*‐[*N*‐succinimidyl)−1,1,3,3‐tetramethyluronium tetrafluoroborate) (Scheme 1). The quinazoline ring of the ARS core was formed via condensation of 2‐amino‐4‐bromo‐5‐chlorobenzoic acid (**5**) and formamidine acetate (**6**). Refluxing quinazolone **7** in POCl_3_ resulted in 4‐chloroquinazoline (**8**) that was transformed to 4‐aminoquinazoline (**9**) in THF saturated by ammonia. This was followed by a Suzuki coupling to form the biaryl skeleton (**11**), and finally we acylated the free amino group with the activated heterocyclic ester (**4**). The final product was purified via preparative RP‐HPLC, and the structures were confirmed by ^1^H and ^13^C NMR spectroscopy and mass spectrometry.

### 2.3 ARS‐F1 Shows Kras^G12C^ Binding, In Vitro and Cellular Activity

**Figure 4:**
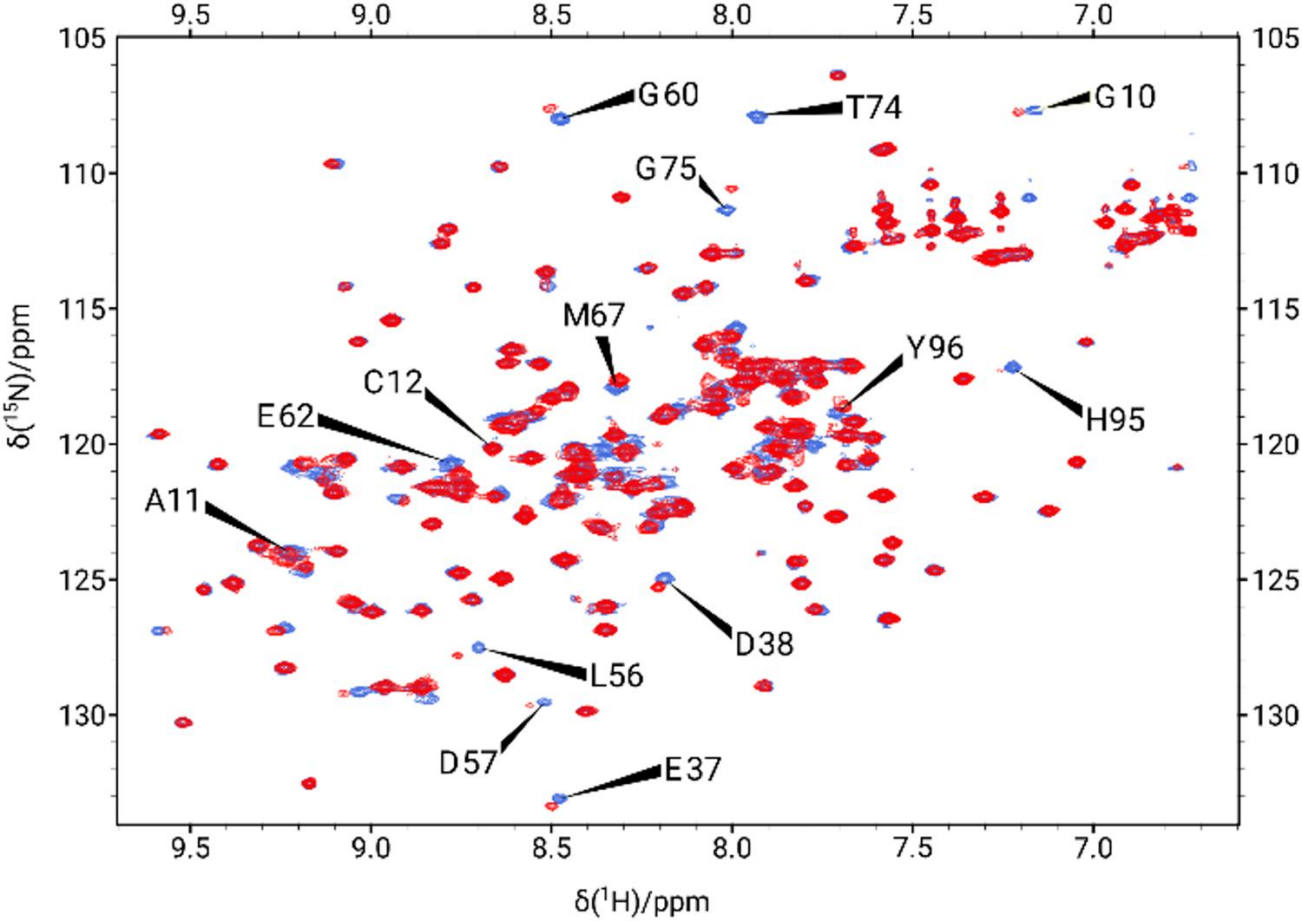
HSQC nuclear magnetic resonance (NMR) spectra of the labeled (red) and unlabeled (blue) KRas^G12C^ protein.

Our first aim was to determine whether **ARS‐F1** binds to the target protein, KRas^G12C^. Therefore, we performed intact MS and enzymatic digestion by Trypsin‐LysC mixture followed by MS/MS measurements. These measurements confirmed that **ARS‐F1** forms covalent bond with the targeted cysteine (C12) (see Supporting Information). Next, we investigated the non‐covalent contacts by HSQC NMR and therefore we incubated the target by hundred‐fold excess of **ARS‐F1**. The assigned spectrum of the free protein [29] was compared with the spectra of the ARS‐853, ARS‐1620 and **ARS‐F1** treated KRas^G12C^ (Figure 4), and from the chemical shift perturbation (CSP) the affected amino acids could be determined (see Supporting Information). The greatest CSPs were observed around the binding pocket (G10, A11, C12, G13, G60, E62, Y96), indicating that **ARS‐F1** binds to the Switch II pocket in a manner similar to the reference inhibitors. Consistent with these observations, molecular docking of **ARS‐F1** revealed a binding pose nearly identical to the experimentally determined structure of ARS‐1620 (Figure 3). In particular, the common scaffold adopts a highly similar orientation within the Switch II pocket, positioning the covalent warhead toward C12 while preserving key interactions with residues such as H95 and T96. The close agreement between the MS, NMR, and docking results strongly supports the proposed binding mode of **ARS‐F1**.

Next, we tested the functional activity of the **ARS‐F1** using a nucleotide exchange assay [30] and cellular assays. In theory, the inhibitor bound to C12 at the allosteric site can block the activation of the protein. In the assay, the inactive mant‐GDP‐bound protein was first incubated with the small molecule for 60 min, then GTP was added to see whether **ARS‐F1** can prevent the protein from its activation in the presence of SOS1 (Son of Sevenless guanine nucleotide exchange factor). In the experiments we used a GTP analog, GppNHp, which cannot be hydrolyzed to GDP by KRas. Based on concentration dependent measurements the IC_50_ was determined to be 35.3 ± 7.2 µM (see  Supporting Information).

The cellular efficacy and selectivity of **ARS‐F1** was assessed on KRas^G12C^ (H1792) and KRas^WT^ (LCLC103H) expressing cells. The cells were treated with the synthesized inhibitor in three replicates per concentration in three independent experiments. The cells were then incubated at 37°C for 72 h and IC_50_ values were determined (Table 3). **ARS‐F1** showed modest, but comparable potency and selectivity to that of ARS‐1620 nominating it as an early stage lead for further development.

**Table 3:** Cellular efficacy and selectivity of ARS‐F1 and ARS‐1620 on cancer cell lines expressing KRas^G12C^ (H1792) and KRas^WT^ (LCLC103H).

Cell line	IC_50_ (µM)		Selectivity	
	ARS‐F1	ARS‐1620	ARS‐F1	ARS‐1620
LCLC103H (WT)	35.0	20.2	—	—
H1792 (G12C)	21.7	10.2	1.6	1.9

## 3 Conclusions

We screened a library of heterocyclic acrylamide bioisosteres against KRas^G12C^. Primary hits were identified by a colorimetric thiol assay and were prioritized by their GSH reactivity, labeling efficiency and specificity assessed by RapidFire MS. The best fragment was a vinylpyrimidine (**F1**) that was first combined *in silico* with the quinazoline core of ARS‐1620 and its docking supported appropriate positioning at the targeted site of KRas^G12C^. Vinylpyridine can be considered as a more stable alternative to the acrylamide warhead against retro‐Michael reaction providing a more stable covalent adduct. After the synthesis of **ARS‐F1**, we evaluated its activity and selectivity. Its binding site was confirmed via intact MS, enzymatic digestion and HSQC NMR measurements. The biological activity was first tested in nucleotide exchange assay and then in vitro in cell viability assay. The activity of **ARS‐F1** was similar to that of ARS‐1620, suggesting that screening heterocyclic acrylamide bioisosteres can provide reasonable starting points for developing new targeted covalent inhibitors.

## 4 Experimental

### 4.1 Chemistry

#### 4.1.1 General

NMR measurements were performed on a System 500 NMR spectrometer (Varian, Palo Alto, CA, USA) or a Varian System 300 spectrometer. ^1^H‐ and ^13^C‐NMR spectra were measured at room temperature (25°C) in an appropriate solvent. ^1^H and ^13^C chemical shifts are expressed in parts per million (δ) referenced to TMS or residual solvent signals. All chemicals and solvents were used as purchased. HPLC‐MS measurements were performed using a LC‐MS‐2020 device (Shimadzu, Kyoto, Japan) equipped with a Reprospher 100 C18 (5 µm, 100 × 3 mm) column and positive‐negative double ion source (DUIS±) with a quadrupole mass spectrometer in a range of 50–1000 m/z. Samples were eluted with gradient elution using eluent A (0.1% formic acid in water) and eluent B (0.1% formic acid in acetonitrile). Flow rate was set to 1.5 mL/min. The initial condition was 0% B eluent, followed by a linear gradient to 100% B eluent by 2 min, from 2 to 3.75 min 100% B eluent was retained, and from 3.75 to 4.5 min back to initial condition and retained to 5 min. The column temperature was kept at 30°C and the injection volume was 1 µL. High resolution mass spectrometric measurements were performed using a Q‐TOF Premier mass spectrometer (Waters, Milford, MA, USA) in positive electrospray ionization mode. Known compounds are characterized by 1H NMR, new compounds are characterized by ^1^H and ^13^C NMR and HRMS. All compounds were of > 95% purity. Benchmark proteins were commercially available: Lysozyme from hen egg: Sigma Aldrich, 62970‐1G‐F, #BCBV6782, 12650‐88‐3; Myoglobin: Sigma Aldrich, M1882‐250mg, #SLB8560V; Carbonic anhydrase II: Sigma Aldrich, C2522‐5mg, #SLBV8282.

#### 4.1.2 Synthesis of ARS‐F1

##### 4.1.2.1 Methyl 5‐vinylpyrimidine‐2‐carboxylate (2)

Methyl 5‐bromopyrimidine‐2‐carboxylate (**1**) (2.38 g, 10.97 mmol) was dissolved in 45 mL dry THF, then Pd(PPh_3_)_2_Cl_2_ (0.77 g, 1.088 mmol) and tributyl‐vinyl‐tin (5.144 mL, 17.6 mmol) was added. The resulting mixture was stirred in a sealed tube for 2 h at 110°C, then the mixture was filtered through celite pad. The product was purified via reverse phase flash chromatography, yielding 1.44 g (80%) pure product. ^1^H‐NMR (300 MHz, DMSO‐*d6*) δ 9.10 (s, 2H), 6.91–6.74 (m, 1H), 6.27 (d, *J* = 17.8 Hz, 1H), 5.65 (d, *J* = 11.1 Hz, 1H), 3.91 (s, 3H).

##### 4.1.2.2 5‐Vinylpyrimidine‐2‐carboxylic acid (3)

Methyl 5‐vinylpyrimidine‐2‐carboxylate (**2**) (1.0 g, 6.090 mmol) was dissolved in 150 mL distilled water, then 9.3 mL 10% HCl solution was added. The resulting mixture was stirred at 60°C for 6 days, then the solvents were evaporated, yielding 0.869 g (95%) pure product. ^1^H‐NMR (300 MHz, DMSO‐*d6*) δ 9.07 (s, 2H), 6.82 (dd, *J* = 17.8, 11.2 Hz, 1H), 6.24 (d, *J* = 17.8 Hz, 1H), 5.62 (d, *J* = 11.2 Hz, 1H).

##### 4.1.2.3 *N*‐[6‐chloro‐7‐(2‐fluoro‐6‐hydroxyphenyl)quinazolin‐4‐yl]−5‐vinylpyrimidine‐2‐carboxamide (4)

5‐Vinylpyrimidine‐2‐carboxylic acid (**3**) (0.013 g, 0.087 mmol) was dissolved in 1 mL MeCN, then TSTU (0.02 g, 0.066 mmol) and DIPEA (0.012 mL, 0.069 mmol) were added, and the resulting mixture was stirred at toom temperature for 30 min. Due to stability issues, compound 4 was used in the reaction with compound 11 without further purification.

##### 4.1.2.4 7‐Bromo‐6‐chloroquinazolin‐4(3*H*)‐one (7)

2‐Amino‐4‐bromo‐5‐chlorobenzoic acid (**5**) (5 g, 19.96 mmol) was dissolved in ethanol (110 mL), then formamidine acetate (6) (20.78 g, 199.60 mmol) was added followed by 16 h stirring at 78°C. The solvent was evaporated, the crude product was rinsed with water, filtered, and dried, yielding the product as a light brown solid (5.05 g, 97%). ^1^H‐NMR (300 MHz, DMSO‐*d6*) δ 12.50 (s, 1H), 8.154, (s, 1H), 8.148 (s, 1H), 8.05 (s, 1H).

##### 4.1.2.5 7‐Bromo‐4,6‐dichloroquinazoline (8)

In a heated flask 7‐bromo‐6‐chloroquinazolin‐4(3*H*)‐one (**7**) (5.05 g 19.46 mmol), POCl_3_ (45 mL) and DIPEA (3.4 mL) were measured followed by 16 h stirring at 106°C. POCl_3_ was distilled, then toluene (20 mL) was added and evaporated two times. The crude product was purified with flash chromatography (eluent: hexane:EtOAc using gradient elution), yielding **8** as a white solid (4.43 g, 82%). ^1^H‐NMR (300 MHz, DMSO‐*d6*) δ 8.21 (s, 1H), 8.16 (s, 1H), 8.06 (s, 1H).

##### 4.1.2.6 7‐Bromo‐6‐chloroquinazolin‐4‐amine (9)

7‐Bromo‐4,6‐dichloroquinazoline (**8**) (1 g, 3.6 mmol) was dissolved in 80 mL THF saturated with ammonia, the resulting mixture was stirred at room temperature for 1 h. Then the solvents were evaporated under reduced pressure. The product was purified via flash chromatography (DCM to MeOH using gradient elution) yielding 0.88 g (95%) pure product. ^1^H‐NMR (300 MHz, DMSO‐*d6*) δ 8.60 (s, 1H), 8.40 (s, 1H), 8.06 (s, 2H), 7.27 (s, 1H).

##### 4.1.2.7 2‐(4‐Amino‐6‐chloroquinazolin‐7‐yl)−3‐fluorophenol (11)

7‐bromo‐6‐chloroquinazolin‐4‐amine (**9**) (0.3 g, 1.16 mmol) were dissolved in 30 mL dioxane:water 4:1, then Na_2_CO_3_ (0.49 g, 4.64 mmol), (2‐fluoro‐6‐hydroxyphenyl)boronic acid (**10**) (0.45 g, 2.90 mmol), RuPhos‐Pd‐G3 (0.19 g, 0.23 mmol) and SPhos (0.095 g, 0.23 mmol) were added. The resulting mixture were stirred at 80°C for 1 h. The solvents were evaporated using reduced pressure. EtOAc was added, and the suspension was filtered through celite pad, the filtrate was concentrated under vacuum. The crude product was purified via flash column chromatography (DCM to MeOH using gradient elution), yielding the product as a yellow solid (0.33 g, 97%). ^1^H‐NMR (300 MHz, DMSO‐*d6*) δ 10.10 (s, 1H), 8.54 (s, 1H), 8.49 (s, 1H), 8.19 (s, 2H), 7.62 (s, 1H), 7.29 (td, *J* = 8.3, 6.9 Hz, 1H), 6.84 (d, *J* = 8.3 Hz, 1H), 6.76 (t, *J* = 8.6 Hz, 1H). ^13^C‐NMR (75 MHz, DMSO‐*d6*) δ 161.13, 158.20, 156.23 (d, *J* = 6.8 Hz), 155.48, 146.69, 136.64, 130.86, 130.43 (d, *J* = 10.9 Hz), 130.11, 123.79, 114.45, 113.37 (d, *J* = 18.7 Hz), 111.68 (d, *J* = 2.8 Hz), 105.62 (d, *J* = 21.9 Hz). Exact Mass [M + H]^+^: Calcd: 290.049 Found: 290.0485.

##### 4.1.2.8 *N*‐[6‐Chloro‐7‐(2‐fluoro‐6‐hydroxyphenyl)quinazolin‐4‐yl]−5‐vinylpyrimidine‐2‐carboxamide (ARS‐F1)

11 (0.05 g, 0.17 mmol) was dissolved in 5 mL dioxane, then 4 (0.052 g, 0.35 mmol) and DIPEA (63 µL, 0.35 mmol) were added. The resulting mixture was stirred overnight at 90°C. The solvents were evaporated, then the crude mixture was purified via prep‐HPLC, resulting in 11 mg pure product (15%). ^1^H‐NMR (300 MHz, DMSO‐*d6*) δ 9.01 (s, 2H), 8.46 (s, 1H), 8.34 (s, 1H), 7.91 (bs, 1H), 7.67 (td, *J* = 8.4, 6.5 Hz, 1H), 7.58 (s, 1H), 7.41 (t, *J* = 7.8 Hz, 2H), 6.75 (dd, *J* = 17.8, 11.2 Hz, 1H), 6.22 (d, *J* = 17.8 Hz, 1H), 5.62 (d, *J* = 11.1 Hz, 1H). ^13^C‐NMR (75 MHz, DMSO‐*d6*) δ 161.26, 156.73, 155.62, 153.72, 149.39, 148.41, 134.29, 133.02, 131.67, 131.56, 131.17, 129.99, 124.41, 122.30, 120.34, 120.12, 119.48, 115.46, 114.30, 114.01, 97.38. Exact Mass [M + H]^+^: Calcd: 422.0814, Found: 422.0831.

## 5 Methods

### 5.1 Expression and Purification of KRas^G12C^

His‐tagged and N‐terminal TEV protease cleavage site added K‐Ras^4B‐G12C^ mutant coded gene contained by pET‐15b expression plasmids. The plasmids were transferred into BL21(DE3) host cells, then grown on 37°C in 2YT nutrient solution, with 180 rpm shaking, using 0.1 g/dm^3^ ampicillin. When OD600 reached 0.7–0.8, the cells were centrifuged, washed with minimal nutrient solution and resuspended (in case of ^15^N‐labeled proteins 1 g/dm^3 15^NH_4_Cl and glucose). After 1 h shaking, cells were induced with 0.5 mM IPTG (isopropyl‐β‐D‐1‐tiogalactopyranoside). After 3 h of 37°C shaking cells were centrifuged, then resuspended in the lysis buffer (500 mM NaCl, 20 mM imidazole, 20 mM Tris, pH = 8.0) and were stored at −20°C until application. The melted cells were exposed by sonication, and the supernatant has been centrifuged. The target protein was separated in three chromatographic steps from the impurities. First nickel nitriloacetic acid (5 mL, Ni‐NTA) metal ion affinity chromatography was used. Lysis buffer was applied to balance the column and after washing we eluted the target protein with a lysis buffer containing 250 mM imidazole. The samples purified this way were dialyzed in a dialysis buffer (500 mM NaCl, 20 mM imidazole, 1 mM DTT, 0.5 mM EDTA, 20 mM Tris, pH = 8.0), while it was digested using His‐tagged TEV protease. Results of cleaning and digestion was checked with sodium dodecyl sulfate with polyacrylamide gel electrophoresis (SDS‐PAGE). The TEV‐digested protein was isolated using Ni‐NTA column to ensure isolation of TEV Protease and HIS‐tag from the protein. Finally, size exclusion chromatography was executed using Superdex 75 Increase (10/300 GL) column in PBS (137 mM NaCl, 2.7 mM KCl, 10 mM Na_2_HPO_4_, 2 mM KH_2_PO_4_, pH = 7.4) containing 10 mM EDTA as eluent. The resulting high purity protein solution was concentrated to 0.5–1.0 mM concentration, then it was frozen in liquid nitrogen and stored at −80°C.

### 5.2 Colorimetric Free Thiol Assay

2 μM GDP‐bound KRas^G12C^ was incubated in the presence of 200 μM of fragments (5% final DMSO concentration) for 1 h at room temperature. Then 4 μL of thiol detection reagent (Invitrogen, M30550 Component A) was added 16 μL of the samples in a black, 384‐well assay plates (Corning, Ref No.: 4514) and the samples were mixed by brief shaking. After incubation the plates in dark at room temperature for 30 min the fluorescence was measured in a microplate reader (BioTek Synergy Mx; *λ*_ex_ = 390 nm and *λ*_em_ = 510 nm). Samples were measured as duplicates and each plate contained a protein sample treated with 200 μM ARS‐853 and another to which 5% DMSO was added. Reactivity of the reference compound was considered 100%, while the protein in 5% DMSO was considered as the baseline (0% reactivity).

### 5.3 Intact Protein LC‐MS

Protein samples were desalted and purified using Amicon Ultra‐0.5 mL Centrifugal Filter units (10 kDa, Merck Millipore) and LC‐MS grade water. Reverse‐phase chromatography‐mass spectrometry (RPLC‐MS) of the intact protein samples were performed on a Waters Acquity BEH300 C4 UPLC column (2.1 × 150 mm, 1.7 µm) under the following parameters: mobile phase “A”: 0.1% trifluoroacetic acid in water, mobile phase “B”: 0.1% trifluoroacetic acid in acetonitrile; flow rate: 400 µL/min; column temperature: 65°C; gradient: 1 min: 5% B, 20 min: 50% B, 21 min: 90% B. UV detection was performed at 220 and 280 nm. The *m/z* range was 400–2000. 10 µL protein sample (approx. 50 pmol protein) was injected onto the column. Deconvolution was performed by MaxEnt 1 software. In case of ARS‐F1 40 µL of the protein in 1 mg/mL concentration was treated with 0.4 µL 50 mM DMSO solution of ARS‐F1 and incubated for 1 h at 37°C. Intact protein analysis and peptide mapping were performed on a high‐resolution hybrid quadrupole‐time‐of‐flight mass spectrometer (Waters Select Series Cyclic IMS, Waters Corp., Wilmslow, UK). The mass spectrometer operated in positive V mode. Leucine enkephalin was used as Lock Mass standard. Chromatographic separations were performed on a Waters Acquity I‐Class UPLC system, coupled directly to the mass spectrometer.

### 5.4 RapidFire MS

The concentration of each protein was 1 μM, except lysozyme that was used in 5 μM due to poor ionization in the MS. The fragments were used in 50 μM, 200 μM, and 500 μM concentrations. Labeling was carried out using in Greiner 384 low volume white plates (#784075). The plates were placed on ice. 50 μL of protein stock solution (1 μM/5 μM lysozyme) was dispensed into wells containing fragment and incubated at room temperature for 24 h. The plates were then frozen at −80°C and sent for analysis by RapidFire MS. An Agilent RapidFire 360 microfluidic system equipped with three 1100 isocratic pumps and a RapidFire C4 Type A solid‐phase extraction (SPE) cartridge (G9203‐80103) was used for sample introduction. Samples were sequentially analyzed by aspirating with vacuum for 250 ms to fill the sample loop (10 μL) directly from a 384‐well microtiter plate. Samples were loaded onto the SPE cartridge and washed using 0.1% (*v/v*) formic acid (solvent A) at a flow rate of 1.50 mL/min for 3500 ms and eluted using 40% acetonitrile in water (v/v) with 0.1% (v/v) formic acid (solvent B) at a flow rate of 1.0 mL/min for 4000 ms. Concurrent with sample elution, the sample loop was washed with solvent B at a flow rate of 1.25 mL/min. The system was re‐equilibrated using solvent A at a flow rate of 1.5 mL/min for 500 ms. Sample needle washes were performed between injections and blank washes performed after each plate row of samples. The entire sampling cycle was approximately 12 s per well enabling the analysis of a 384‐well plate in approximately 77 min. Mass spectrometric data were acquired on an Agilent 6220 time‐of‐flight (ToF) MS system, operated with a dual electrospray ionization (ESI) source ion source, in positive ionization mode. The instrument parameters were as follows: gas temperature 300°C, drying gas 13 L/min, nebulizer 45 psi, capillary 5.0 kV, fragmentor 290 V, skimmer 65 V, and octopole RF 300 V peak‐to‐peak. Data were acquired at the rate of 5 spectra/s. The mass range was calibrated using the Agilent positive ion tune mix, over the m/z range 300–3200. Agilent RapidFire v4.0 and Agilent MassHunter B.04.00 software were employed for data acquisition and processing. The total ion chromatograms (TIC) were extracted (region containing protein) and the summed scans were deconvoluted over a *m/z* range with an expected mass range dependent on the protein. Raw data was further analyzed by an R Studio script.

### 5.5 Peptide Mapping

The modification site was determined by proteolysis and RPLC‐MS peptide mapping. Briefly, the protein was enzymatically digested after buffer exchange using Amicon Ultra‐0.5 mL Centrifugal Filter units (10 kDa, Merck Millipore). Trypsin‐LysC mixture (Promega Corporation, Madison, USA) was used for the enzymatic digestions. Protein sample was reduced by dithiothreitol at 37°C for 30 min. After reduction, the protein was digested using 1:50 enzyme:protein ratio. Overnight digestion was performed by Trypsin‐LysC mixture in 50 mM NH_4_HCO_3_ solution at 37°C. Tryptic digestion was stopped by adding formic acid in a final concentration of 0.2% (V/V). After digestion, the reduction with dithiothreitol was repeated with a short incubation (5 min, 37°C). Gradient elution of the peptides was performed on a Waters Acquity CSH Peptide C18 UPLC column (2.1 × 150 mm, 1.7 µm) under the following parameters: mobile phase “A”: 0.1% formic acid in water, mobile phase “B”: 0.1% formic acid in acetonitrile; flow rate: 300 µL/min; column temperature: 60°C; gradient: 2 min: 2% B, 80 min: 45% B, 81 min: 85% B. MS^E^ experiment was performed using collision voltage ramping under the following parameters: *m/z* 50–2000, scan time: 0.3 s, single Lock Mass: leucine enkephalin; low energy: 6 V, high energy: ramping 19‐45 V. BiopharmaLynx 1.3.5 software (Waters Corp., Wilmslow, UK) was used for data analysis.

### 5.6 HSQC NMR Spectroscopy

NMR samples contained 70 μM ^15^N‐labeled GDP‐bound KRas^G12C^ protein, 100–600 μM binding partner, 10 mM MgCl_2_, 3 mM NaN_3_, in PBS buffer, 5% DMSO‐*d_6_*, 7% D_2_O, 0.5% DSS. The samples were measured twice, immediately after the sample preparation and 1 day later, in between they were stored at 4°C–8°C. ^1^H,^15^N‐SOFAST‐HMQC (fast version of ^1^H,^15^N‐HSQC) NMR spectra were acquired at 298 K on a Bruker AVANCE III spectrometer operating at 700.05 MHz for ^1^H and 70.94 MHz for ^15^N, equipped with a 5 mm Prodigy TCI H&F‐C/N‐D, z‐gradient probehead. Temperature was calibrated by standard methanol solution. The chemical shifts were referenced with respect to the ^1^H‐resonance of internal DSS standard, while ^15^N chemical shifts were referenced indirectly via the gyromagnetic ratios according to the IUPAC conventions. All NMR data were processed with TopSpin 3.5 and analyzed in NMRFAM‐SPARKY [31].

### 5.7 Nucleotide Exchange Assay

KRas^G12C^ protein was first buffer exchanged into low magnesium buffer (20 mM HEPES‐NaOH (pH 7.5), 50 mM NaCl, 0.5 mM MgCl_2_) using a NAP5 column (catalog no.: 17‐0583‐1, Cytiva). The proteins were then incubated with 20‐fold molar excess of *N*‐methylanthraniloyl (MANT)‐GDP (catalog no.: 69244, Sigma‐Aldrich) in loading buffer (50 mM NaCl, 20 mM HEPES‐NaOH [pH 7.5], 0.5 mM MgCl_2_, 10 mM EDTA, and 1 mM dithiothreitol (DTT)) in a total volume of 200 μL at 20°C for 90 min. The reaction was stopped by adding MgCl_2_ to a final concentration of 10 mM, then incubated at 20°C for 30 min. The unbound MANT‐GDP was removed using the NAP‐5 column equilibrated with nucleotide exchange buffer (40 mM HEPES‐NaOH (pH 7.5), 50 mM NaCl, 10 mM MgCl_2_, 2 mM dithiothreitol DTT). First, the MANT‐GDP bound KRAS^G12C^ protein (in a final 1 μM molar concentration) was incubated with the inhibitor molecules for 60 min. After the incubation, the MANT‐GDP KRAS‐inhibitor mixtures were loaded into a black 384 well microplate in the presence or absence of the inhibitor molecule in various concentrations. The nucleotide exchange reaction was initiated by adding 100 fold molar excess of GppNHp (catalog no.: G0635, Sigma‐Aldrich), a non‐hydrolysable GTP analog and the SOS1 exchange domain (catalog no.: GE02, Cytoskeleton Inc.) protein in 0.2 μM final concentration. The change in fluorescence intensity was measured every 45 s in room temperature for 90 min on an EnvSpire plate reader (PerkinElmer Inc.). The measured values were fitted to a single exponential function by using GraphPad Prism 8 software.

### 5.8 Cell Proliferation Assay

H1792 (KRas^G12C^, lung cancer) cell line was obtained from the American Type Culture Collection (ATCC), and LCLC‐103H (KRas^WT^) cell line was obtained from the German collection of microorganisms and cell cultures (DSMZ). Cells were cultured in sterile T25 or T75 flasks with ventilation cap (Sarstedt, Nümbrecht, Germany) at 37°C in a humidified atmosphere with 5% CO_2_. On the first day the cell lines were seeded in density 5000 cells per well in 5% FBS (serum) containing RPMI‐1640, supplemented with 1% penicillin/streptomycin. On the second day, after 24 h of incubation on 37°C, cells were treated with various concentrations of **ARS‐F1** and ARS‐1620 from 20 mM DMSO stocks, and incubated on 37°C for 72 h. Cells were treated with compounds at concentrations: 100 µM, 20 µM, 4 µM, 800 nM, 160 nM, and 32 nM (dilution factor 5x), dissolved in serum free medium. Final concentration of serum was 2.5%. 25 µM and 2.5 µM concentrations were prepared independently. Control wells were treated with serum free growth medium, and DMSO. Final concentration of serum was 2.5%, final concentration of DMSO was 0.5%). Three replicates per concentration and three independent experiments were performed. Cells under treatment were incubated at 37°C, 5% CO_2_ for 72 h. Cell viability was determined by MTT assay, IC_50_ was calculated using GraphPad Prism Software.

### 5.9 GSH Assay Based on HPLC‐MS

A 250 μM solution of the fragment [in PBS buffer (pH 7.4) with 5% acetonitrile] with a 100 μM solution of indoprofen as the internal standard was incubated with 5 mM glutathione. The reaction mixture was analyzed by HPLC‐MS sampling after 0, 1, 2, 4, 8, 12, 24, 48, and 72 h. The AUC (area under the curve) values were determined via integration of HPLC or MS chromatograms and then corrected with the internal standard. The fragments' AUC values were subjected to ordinary least‐squares (OLS) linear regression, and to compute the important parameters (kinetic rate constant and half‐life time), an Excel sheet was applied. The data are expressed as means of duplicate determination. The kinetic rate constant for the degradation and corrected GSH reactivity were calculated as follows. The reaction half‐life for pseudo‐first‐order reactions (*t*_1/2_) is ln 2/*k*, where *k* is the reaction rate.

### 5.10 Molecular Docking

#### 5.10.1 Induced Fit Docking

X‐ray structure of KRas^G12C^ in complex with ARS 1620 (PDBID: 5V9U) was used to perform the docking simulations. Protein was prepared with Protein Preparation Wizard (Schrödinger Release 2021‐3) using default methods. Ligands were prepared with Schrödinger Ligprep [32] using default methods. Docking was performed with Schrödinger Induced Fit Docking [33, 34, 35, 36] (Schrödinger Release 2021‐3), generating 20 possible binding conformations. Redocking was done into structures within 30 kcal/mol of the best structure, and within the top 20 structures overall using single precision method. Proteins with the best ligand binding conformations were selected for covalent docking.

#### 5.10.2 Covalent Docking

CovDock of Schrödinger (Schrödinger Release 2021‐3) [37] was used to perform the covalent docking. Proteins were prepared as described previously. After choosing the appropriate reaction type default settings were used.

## Conflicts of Interest

The authors declare no conflicts of interest.

## Supporting information


Supporting File

## Data Availability

The data that supports the findings of this study are available in the supporting material of this article.

## References

[1] C. M. Marshall , J. G. Federice , C. N. Bell , P. B. Cox , and J. T. Njardarson , “An Update on the Nitrogen Heterocycle Compositions and Properties of U.S. FDA‐Approved Pharmaceuticals (2013–2023),” Journal of Medicinal Chemistry 67 (2024): 11622–11655. 10.1021/acs.jmedchem.4c01122 38995264

[2] N. Pemberton , N. Compagne , A. Argyrou , et al., “Vinylpyridine as a Tunable Covalent Warhead Targeting C797 in EGFR,” ACS Medicinal Chemistry Letters 15 (2024): 583–589. 10.1021/acsmedchemlett.3c00425 PMC11089552 38746885

[3] M. Schwarz , M. Kurkunov , F. Wittlinger , et al., “Development of Highly Potent and Selective Covalent FGFR4 Inhibitors Based on SNAr Electrophiles,” Journal of Medicinal Chemistry 67 (2024): 6549–6569. 10.1021/acs.jmedchem.3c02483 38604131

[4] E. Karaj , S. H. Sindi , N. Kuganesan , L. Perera , W. Taylor , and L. M. V. Tillekeratne , “Tunable Cysteine‐Targeting Electrophilic Heteroaromatic Warheads Induce Ferroptosis,” Journal of Medicinal Chemistry 65 (2022): 11788–11817. 10.1021/acs.jmedchem.2c00909 PMC10408038 35984756

[5] H.‐R. Kim , D. P. Byun , K. Thakur , et al., “Discovery of a Tunable Heterocyclic Electrophile 4‐Chloro‐pyrazolopyridine That Defines a Unique Subset of Ligandable Cysteines,” ACS Chemical Biology 19 (2024): 1082–1092. 10.1021/acschembio.4c00025 PMC11107811 38629450

[6] A. Keeley , P. Ábrányi‐Balogh , M. Hrast , et al., “Heterocyclic Electrophiles as new MurA Inhibitors,” Arch Pharm 351 (2018): 1800184. 10.1002/ardp.201800184. 30461051

[7] A. Keeley , P. Ábrányi‐Balogh , and G. M. Keserű , “Design and Characterization of a Heterocyclic Electrophilic Fragment Library for the Discovery of Cysteine‐Targeted Covalent Inhibitors,” MedChemComm 10 (2019): 263–267. 10.1039/c8md00327k PMC6390469 30881613

[8] A. B. Keeley , A. Kopranovic , V. Di Lorenzo , et al., “Electrophilic Minifrags Revealed Unprecedented Binding Sites for Covalent HDAC8 Inhibitors,” Journal of Medicinal Chemistry 67 (2024): 572–585. 10.1021/acs.jmedchem.3c01779 PMC10788917 38113354

[9] P. Ábrányi‐Balogh , A. Keeley , G. G. Ferenczy , et al., “Next‐Generation Heterocyclic Electrophiles as Small‐Molecule Covalent Mura Inhibitors,” Pharmaceuticals 15 (2022): 1484. 10.3390/ph15121484 PMC9781958 36558935

[10] R. Sebastiano , A. Citterio , M. Lapadula , and P. G. Righetti , “A New Deuterated Alkylating Agent for Quantitative Proteomics,” Rapid Communications in Mass Spectrometry 17 (2003): 2380–2386. 10.1002/rcm.1206 14587083

[11] J. Andrew , J. M. Sharpe , M. Wallace , et al., “Synthesis and Evaluation of 4‐(α‐arylvinyl)pyridines as Activated Linker Groups for Application in Antibody–Drug Conjugates,” Organic & Biomolecular Chemistry 24 (2026): 5679–5684. 10.1039/d6ob00614k PMC13307478 42359911

[12] H. Seki , S. J. Walsh , J. D. Bargh , J. S. Parker , J. Carroll , and D. R. Spring , “Rapid and Robust Cysteine Bioconjugation With Vinylheteroarenes,” Chemical Science 12 (2021): 9060–9068. 10.1039/d1sc02722k PMC8261766 34276935

[13] S. J. Walsh , S. Omarjee , W. R. J. D. Galloway , et al., “A General Approach for the Site‐Selective Modification of Native Proteins, Enabling the Generation of Stable and Functional Antibody–Drug Conjugates,” Chemical Science 10 (2019): 694–700. 10.1039/c8sc04645j PMC6349026 30774870

[14] Y. Li , S. Fu , J. Yang , et al., “Acrylamide Bioisosterism: Alkenyl Aromatic Heterocycles as Reactivity‐Tunable Warheads for Covalent BTK Inhibitors,” Journal of Medicinal Chemistry 69 (2026): 5828–5850. 10.1021/acs.jmedchem.5c03212 41735169

[15] M. P. Patricelli , M. R. Janes , and L.‐S. Li , “Selective Inhibition of Oncogenic KRAS Output With Small Molecules Targeting the Inactive State,” Cancer Discovery 6 (2016): 316–329. 10.1158/2159-8290.CD-15-1105 26739882

[16] M. R. Janes , J. Zhang , L.‐S. Li , et al., “Targeting KRAS Mutant Cancers With a Covalent G12C‐Specific Inhibitor,” Cell 172 (2018): 578–589.e17. 10.1016/j.cell.2018.01.006 29373830

[17] J. H. Strickler , H. Satake , T. J. George , et al., “Sotorasib inKRASp.G12C–Mutated Advanced Pancreatic Cancer,” New England Journal of Medicine 388 (2023): 33–43. 10.1056/NEJMoa2208470 PMC10506456 36546651

[18] P. A. Jänne , G. J. Riely , S. M. Gadgeel , et al., “Adagrasib in Non–Small‐Cell Lung Cancer Harboring aKRASG12C Mutation,” New England Journal of Medicine 387 (2022): 120–131. 10.1056/NEJMoa2204619 35658005

[19] L. Huang , Z. Guo , F. Wang , and L. Fu , “KRAS Mutation: From Undruggable to Druggable in Cancer,” Signal Transduction and Targeted Therapy 6 (2021): 386. 10.1038/s41392-021-00780-4 PMC8591115 34776511

[20] B. El Osta , “KRAS G12C Mutation: From Undruggable Target to Potentially Agnostic Biomarker,” Translational Lung Cancer Research 12 (2023): 1147–1151. 10.21037/tlcr-23-174 PMC10326771 37425416

[21] M. Santarpia , G. Ciappina , C. C. Spagnolo , et al., “Targeted Therapies for KRAS‐Mutant Non‐Small Cell Lung Cancer: From Preclinical Studies to Clinical Development—A Narrative Review,” Translational Lung Cancer Research 12 (2023): 346–368. 10.21037/tlcr-22-639 PMC9989806 36895930

[22] N. Péczka , I. Ranđelović , Z. Orgován , et al., “Contribution of Noncovalent Recognition and Reactivity to the Optimization of Covalent Inhibitors: A Case Study on KRasG12C,” ACS Chemical Biology 19 (2024): 1743–1756. 10.1021/acschembio.4c00217 PMC11334105 38991015

[23] G. L. Ellman , “Tissue Sulfhydryl Groups,” Archives of Biochemistry and Biophysics 82 (1959): 70–77. 10.1016/0003-9861(59)90090-6 13650640

[24] C. K. Riener , G. Kada , and H. J. Gruber , “Quick Measurement of Protein Sulfhydryls With Ellman's Reagent and With 4,4′‐Dithiodipyridine,” Analytical and Bioanalytical Chemistry 373 (2002): 266–276. 10.1007/s00216-002-1347-2 12110978

[25] T. Yan , H. S. Desai , L. M. Boatner , S. L. Yen , J. Cao , and M. F. Palafox , “SP3‐FAIMS Chemoproteomics for High‐Coverage Profiling of the Human Cysteinome*,” ChemBioChem 22 (2021): 1841–1851. 10.1002/cbic.202000870 PMC8942723 33442901

[26] E. K. Kemper , Y. Zhang , M. M. Dix , and B. F. Cravatt , “Global Profiling of Phosphorylation‐Dependent Changes in Cysteine Reactivity,” Nature Methods 19 (2022): 341–352. 10.1038/s41592-022-01398-2 PMC8920781 35228727

[27] D. Y. Yoo , A. D. Hauser , S. T. Joy , D. Bar‐Sagi , and P. S. Arora , “Covalent Targeting of Ras G12C by Rationally Designed Peptidomimetics,” ACS Chemical Biology 15 (2020): 1604–1612. 10.1021/acschembio.0c00204 PMC7739374 32378881

[28] M. V. Huynh , D. Parsonage , T. E. Forshaw , et al., “Oncogenic KRAS G12C: Kinetic and Redox Characterization of Covalent Inhibition,” Journal of Biological Chemistry 298 (2022): 102186. 10.1016/j.jbc.2022.102186 PMC9352912 35753348

[29] G. Pálfy , I. Vida , and A. Perczel , “1H, 15N Backbone Assignment and Comparative Analysis of the Wild Type and G12C, G12D, G12V Mutants of K‐Ras Bound to GDP at Physiological Ph,” Biomolecular NMR Assignments 14 (2020): 1–7. 10.1007/s12104-019-09909-7 PMC7069925 31468366

[30] C. H. Gray , J. Konczal , M. Mezna , S. Ismail , J. Bower , and M. Drysdale , “A Fully Automated Procedure for the Parallel, Multidimensional Purification and Nucleotide Loading of the Human GTPases KRas, Rac1 and Ralb,” Protein Expression and Purification 132 (2017): 75–84. 10.1016/j.pep.2017.01.010 PMC5415301 28137655

[31] W. Lee , M. Tonelli , and J. L. Markley , “NMRFAM‐SPARKY: Enhanced Software for Biomolecular NMR Spectroscopy,” Bioinformatics 31 (2015): 1325–1327. 10.1093/bioinformatics/btu830 PMC4393527 25505092

[32] Schrödinger Suite 2021‐3: LigPrep, Schrödinger (LLC, 2021).

[33] R. Farid , T. Day , R. A. Friesner , and R. A. Pearlstein , “New Insights About HERG Blockade Obtained From Protein Modeling, Potential Energy Mapping, and Docking Studies,” Bioorganic & Medicinal Chemistry 14, no. 9 (2006): 3160–3173. 10.1016/j.bmc.2005.12.032 16413785

[34] W. Sherman , T. Day , M. P. Jacobson , R. A. Friesner , and R. Farid , “Novel Procedure for Modeling Ligand/Receptor Induced Fit Effects,” Journal of Medicinal Chemistry 49, no. 2 (2006): 534–553. 10.1021/jm050540c 16420040

[35] W. Sherman , H. S. Beard , and R. Farid , “Use of an Induced Fit Receptor Structure in Virtual Screening,” Chemical Biology & Drug Design 67, no. 1 (2006): 83–84. 10.1111/j.1747-0285.2005.00327.x 16492153

[36] Schrödinger Release 2021‐3: Induced Fit Docking Protocol. Schrödinger (LLC, 2021).

[37] K. Zhu , K. W. Borrelli , J. R. Greenwood , et al., “Docking Covalent Inhibitors: A Parameter Free Approach To Pose Prediction and Scoring,” Journal of Chemical Information and Modeling 54, no. 7 (2014): 1932–1940. 10.1021/ci500118s 24916536

