# Supplementary material for: Fragment‐Based Screening of Heterocyclic Electrophiles Identified Acrylamide Bioisoteres for Covalent KRasG12C Inhibitors
Source: Arch Pharm (Weinheim). 2026 Sep 21;359(9):e70342. doi: 10.1002/ardp.70342 (PMC13594984; doi:10.1002/ardp.70342)
Supplement: Supplementary file 1 [file ARDP-359-e70342-s001.docx]

Supporting Information

**Fragment-based screening of heterocyclic electrophiles identified acrylamide bioisoteres for covalent KRas^G12C^ inhibitors**

Nikolett Péczka^a,b^, Aaron Keeley^a^, Péter Ábrányi-Balogh^a,b^, Zoltán Orgován^a^, László Petri^a^, Emma K. Grant^c^, Chad Quinn^c^, Soma Csanády^a^, Ivan Randelovic^d^, József Tóvári^d^, Jacob Bush^c^ and György M. Keserű^a,b^

^a^HUN-REN Research Centre for Natural Sciences, Medicinal Chemistry Research Group and National Drug Discovery and Development Laboratory, 1117 Budapest, Hungary.

^b^Budapest University of Technology and Economics, Department of Organic Chemistry and Technology, 1111 Budapest, Hungary

^c^GlaxoSmithKline, Gunnels Wood Road, Stevenage, Hertfordshire, SG1 2NY, UK

^d^National Institute of Oncology, Department of Experimental Pharmacology and the National Tumor Biology Laboratory, 1122 Budapest, Hungary

Table of contents

[1.	Supplementary Tables and Figures	2](#_Toc236423403)

[1.1	Supplementary Table S1 Colorimetric free thiol assay results	2](#_Toc236423404)

[1.2	HSQC NMR spectra of ARS-853 (A) and ARS-1620 (B) + KRas^G12C^	3](#_Toc236423405)

[1.3	Intact MS spectrum and digestion result of ARS-F1 labelled KRas^G12C^	4](#_Toc236423406)

[1.4	Exchange assay of ARS-F1	6](#_Toc236423407)

[1.5	NMR spectra of key intermediates and ARS-F1	6](#_Toc236423408)

# 1. Supplementary Tables and Figures

## 1.1 Supplementary Table S1 Colorimetric free thiol assay results

| **Compound No.** | **InChI** | **Free Thiol ratio [%]** |
| --- | --- | --- |
| A1 | InChI=1S/C7H7N/c1-2-7-5-3-4-6-8-7/h2-6H,1H2 | 95 |
| A2 | InChI=1S/C7H5N/c1-2-7-5-3-4-6-8-7/h1,3-6H | 85 |
| B1 | InChI=1S/C7H7N/c1-2-7-4-3-5-8-6-7/h2-6H,1H2 | 93 |
| B2 | InChI=1S/C7H5N/c1-2-7-4-3-5-8-6-7/h1,3-6H | 93 |
| C1 | InChI=1S/C7H7N/c1-2-7-3-5-8-6-4-7/h2-6H,1H2 | 91 |
| C2 | InChI=1S/C7H5N/c1-2-7-3-5-8-6-4-7/h1,3-6H | 26 |
| D2 | InChI=1S/C6H4N2/c1-2-6-7-4-3-5-8-6/h1,3-5H | 105 |
| E1 | InChI=1S/C6H6N2/c1-2-6-3-4-7-5-8-6/h2-5H,1H2 | 84 |
| F1 | InChI=1S/C6H6N2/c1-2-6-3-7-5-8-4-6/h2-5H,1H2 | 25 |
| F2 | InChI=1S/C6H4N2/c1-2-6-3-7-5-8-4-6/h1,3-5H | 103 |
| G1 | InChI=1S/C6H6N2/c1-2-6-5-7-3-4-8-6/h2-5H,1H2 | 54 |
| G2 | InChI=1S/C6H4N2/c1-2-6-5-7-3-4-8-6/h1,3-5H | 70 |
| H2 | InChI=1S/C5H4N2/c1-2-5-6-3-4-7-5/h1,3-4H,(H,6,7) | 73 |
| I1 | InChI=1S/C5H6N2/c1-2-7-4-3-6-5-7/h2-5H,1H2 | 96 |
| I2 | InChI=1S/C6H6N2/c1-2-4-8-5-3-7-6-8/h1,3,5-6H,4H2 | 55 |
| J1 | InChI=1S/C5H6N2/c1-2-5-3-6-4-7-5/h2-4H,1H2,(H,6,7) | 40 |
| K1 | InChI=1S/C5H6N2/c1-2-5-3-4-6-7-5/h2-4H,1H2,(H,6,7) | 91 |
| K2 | InChI=1S/C5H4N2/c1-2-5-3-4-6-7-5/h1,3-4H,(H,6,7) | 102 |
| L1 | InChI=1S/C5H6N2/c1-2-5-3-6-7-4-5/h2-4H,1H2,(H,6,7) | 92 |
| L2 | InChI=1S/C5H4N2/c1-2-5-3-6-7-4-5/h1,3-4H,(H,6,7) | 107 |
| M1 | InChI=1S/C5H6N2/c1-2-7-5-3-4-6-7/h2-5H,1H2 | 108 |
| M2 | InChI=1S/C6H6N2/c1-2-5-8-6-3-4-7-8/h1,3-4,6H,5H2 | 88 |
| N1 | InChI=1S/C11H9NO/c1-2-11-12-8-10(13-11)9-6-4-3-5-7-9/h2-8H,1H2 | 33 |
| O1 | InChI=1S/C7H9NO/c1-4-7-5(2)8-9-6(7)3/h4H,1H2,2-3H3 | 71 |
| O2 | InChI=1S/C7H7NO/c1-4-7-5(2)8-9-6(7)3/h1H,2-3H3 | 109 |
| P2 | InChI=1S/C5H3NS/c1-2-5-3-6-4-7-5/h1,3-4H | 93 |
| Q1 | InChI=1S/C5H5NS/c1-2-5-6-3-4-7-5/h2-4H,1H2 | 21 |

## 1.2 HSQC NMR spectra of ARS-853 (A) and ARS-1620 (B) + KRas^G12C^

**ARS-853**

**ARS-1620**

## 1.3 Intact MS spectrum and digestion result of ARS-F1 labelled KRas^G12C^

Intact protein MS analysis: A.) raw MS spectrum and B.) processed, deconvoluted MS spectrum of the unmodified KRas^G12C^ protein. C.) raw MS spectrum and D.) processed, deconvoluted MS spectrum of the KRas^G12C^ protein labelled with **ARS-F1**.

Data of the identified modification site (a) tryptic peptide, b) ProAlanase digested peptide with **ARS-F1** modification):

a) Sequence: ^8^LVVVGACGVGK^18^; calculated peptide mass: 1421.6481; measured *m/z*: 474.8896, [M+3H]^3+^, mass error: 2.1 ppm, measured mass: 1421.6451 Da, intensity: 542 counts.

Sequence: ^8^LVVVGACGVGK^18^; calculated peptide mass: 1403.6375; measured *m/z*: 1404.6702, [M+H]^+^, mass error: 17.6 ppm, measured mass: 1403.6622 Da, intensity: 1114 counts.

Sequence: ^2^HMTEYKLVVVGACGVG^17^; calculated peptide mass: 2080.8855; measured *m/z*: 694.6415, [M+3H]^3+^, mass error: 7.4 ppm, measured mass: 2080.9009 Da, intensity: 1989 counts.

Sequence: ^2^ HMTEYKLVVVGACGVGKSALTIQL^25^; calculated peptide mass: 3358.4978; measured *m/z*: 672.72, [M+5H]^5+^, mass error: 18.7 ppm, measured mass: 3358.5605 Da, intensity: 1207 counts.

b)

c) Sequence: ^9^VVVGACGVGKSALTIQLIQNHFVDEYDP^36^; calculated peptide mass: 3374.5750; measured *m/z*: 1125.8738, [M+3H]^3+^, mass error: 6.6 ppm, measured mass: 3375.6025 Da, intensity: 934 counts.

## 1.4 Exchange assay of ARS-F1

## 1.5 NMR spectra of key intermediates and ARS-F1

Methyl 5-vinylpyrimidine-2-carboxylate (**2**)

5-Vinylpyrimidine-2-carboxylic acid (**3**)

7-Bromo-6-chloroquinazolin-4(3H)-one (**7**):

7-Bromo-4,6-dichloroquinazoline (**8**):

7-Bromo-6-chloroquinazolin-4-amine (**9**):

2-(4-Amino-6-chloroquinazolin-7-yl)-3-fluorophenol (**11**):

*N*-[6-Chloro-7-(2-fluoro-6-hydroxyphenyl)quinazolin-4-yl]-5-vinylpyrimidine-2-carboxamide (**ARS**-**F1**):
